# Five genome sequences of bacterial environmental isolates from Gilkey Glacier, Juneau Icefield, Alaska

**DOI:** 10.1128/mra.01420-25

**Published:** 2026-07-02

**Authors:** Kathryn E. Caruso, Ashley S. Kleinman, Cheyenne Darrow, Markus Dieser, Heidi J. Smith, Christopher R. Chin, Christopher E. Mason, Christine M. Foreman

**Affiliations:** 1 Center for Biofilm Engineering, Montana State University, Bozeman, Montana, USA; 2 Department of Microbiology and Cell Biology, Montana State University 123776, Bozeman, Montana, USA; 3 Department of Earth, Atmospheric, and Planetary Sciences, Massachusetts Institute of Technology Department of Earth Atmospheric & Planetary Sciences 517829, Cambridge, Massachusetts, USA; 4 Department of Chemical and Biological Engineering, Montana State University 33052, Bozeman, Montana, USA; 5 Department of Medicine, Weill Cornell Medicine 12295, New York, New York, USA; 6 Department of Systems and Computational Biomedicine, Weill Cornell Medicine 12295, New York, New York, USA; 7 The HRH Prince Alwaleed Bin Talal Bin Abdulaziz Alsaud Institute for Computational Biomedicine, Weill Cornell Medicine 12295, New York, New York, USA; 8 The WorldQuant Initiative for Quantitative Prediction, Weill Cornell Medicine 12295, New York, New York, USA; California State University San Marcos, San Marcos, California, USA

**Keywords:** glacier, Alaska, Juneau Icefield

## Abstract

We report the genomic sequences of five environmental bacterial isolates (two *Acinetobacter* spp., *Halpernia* sp., *Glaciimonas* sp., and *Pseudomonas* sp.) from a supraglacial stream on the Gilkey Glacier, Alaska. These isolates provide resources for investigating distribution, survival, and biogeochemical cycling in cryospheric environments.

## ANNOUNCEMENT

Glaciers in the Juneau Icefield, Alaska, are rapidly receding (1), and cataloging their diversity before they are gone is critical (2). Water samples were collected into sterile Nalgene bottles from a supraglacial stream (58.826438°N, −134.322369°W) on the Gilkey Glacier, Alaska. Bacteria were aerobically isolated by spread plating 100 μL liquid directly onto R2A agar plates at 4°C in the dark. Isolates were selected after 40 days and cultured based on colony morphology and pigmentation. The cetyltrimethylammonium bromide procedure was used for extracting DNA from bacterial isolates grown to late exponential phase in R2A broth at 4°C while shaking at 150 rpm (3).

Libraries were prepared using the Oxford Nanopore Technologies (ONT) Native Barcode Kit SQK-NBD114.96 and the PacBio Onso Kit PN: 102-860-300. ONT libraries required no shearing or size selection. Similarly sized libraries were pooled and sequenced in batches for even coverage. PacBio library preparation was done according to the manufacturer's specification, starting with 200 ng DNA. DNA was sheared using PacBio’s fragmentation enzyme at the recommended fragmentation time and temperature. Final library sizes were between 526 and 582 bp. ONT libraries were sequenced on the Oxford Nanopore PromethION FLO-PRO114M, and PacBio libraries were sequenced on PacBio Onso (v01). ONT reads were basecalled (Dorado 0.5.0, model dna_r10.4.1_e8.2_400bps_sup@v4.2.0), which simultaneously performed adapter and barcode trimming during FASTQ generation. PacBio obc2fastq (v5.11) was used to generate fastqs and trim barcodes and adapters from PacBio Onso sequencing. Trimmed reads were quality controlled using FastQC (v0.11.9) (4).

Genomes were assembled using Flye (v2.9.3) (5) with --nano-hq parameters for long-read assemblies and metaSPAdes (v3.15.3) (6, 7) for hybrid assemblies of short- and long-read data. Hybrid assembly was attempted for all samples; long-read-only Flye assemblies were used where hybrid assembly failed. The GG3 Flye assembly was further polished using Medaka (v2.2.0) (8) and Polypolish (v0.6.1) (9). Assembly quality was assessed using QUAST (v5.2.0) (10), and sequencing read quality was assessed using NanoPlot (v1.46.2) (11) for Oxford Nanopore reads and SeqKit (v2.12.0) (12) for Onso reads. The highest quality assembly for each genome was selected for submission.

Taxonomic classification was performed using GTDB-Tk (v2.4.0) (13) with the classify_wf workflow and default parameters, utilizing the GTDB release r220 reference database (14). The Foreign Contamination Screen (FCS) tool suite (15) was used to screen for adaptor contamination. Flagged sequences were removed, and cleaned assemblies were validated using FCS-GX (15). Annotation was performed by NCBI using the Prokaryotic Genome Annotation Pipeline (PGAP) (v6.10) (16). Based on PGAP annotation, all five genomes encode cold shock proteins. Genome completeness and contamination were evaluated by NCBI using CheckM (v1.2.4–v1.2.5) (17) with taxon-specific marker sets; only assemblies meeting completeness >95% and contamination <5% are reported here. Sequence details are presented in Table 1.

**TABLE 1:** Summary of genome characteristics

Organism Accession no. BioSample accession no. Assembly method	Closest ANI Reference species (% ANI)	Size (bp) # Contigs Coverage (×)	Completeness (%) Contamination (%)	Read count (bp)/N50 (ONT, Onso)	N50 (bp)	GC (%) # CDS genes
*Acinetobacter* sp. ANC 3781 strain GG2 JBRIIS000000000 SAMN51801132 Flye	GCF_004331285.1 *Acinetobacter* sp004331285 (98.35)	3,539,468 5 251	99.79 0.71	ONT: 35,709/9,194	3,193,273	40.17 3,282
*Halpernia* sp. GG3 JBRIIR000000000 SAMN51801133 Flye, Medaka, Polypolish	GCF_900113805.1 *Halpernia frigidisoli* (80.56)^*a*^	2,655,472 17 100	97.44 1.49	ONT: 15,297/10,714	409,605	33.27 2,469
*Glaciimonas* sp. GG7 JBRIIQ000000000 SAMN51801134 SPAdes hybrid	No reliable ANI match	4,534,362 270 328	98.14 0.99	ONT: 151,659/8,740 Onso: 207,562/150	39,782	51.44 4,213
*Pseudomonas* sp. GG8 JBRIIP000000000 SAMN51801135 Flye	GCF_900095225.1 *Pseudomonas_E* sp900095225 (94.2)^*b*^	5,944,138 26 80	98.56 2.61	ONT: 69,500/12,076	5,673,401	57.17 5,483
*Acinetobacter* sp. GG23 JBYMDJ000000000 SAMN56310562 SPAdes hybrid	GCF_004331285.1 *Acinetobacter* sp004331285 (98.33)	3,501,636 36 88	99.90 0.62	ONT: 14,345/9,728 Onso: 296,925/150	249,818	40.09 3,282

^*a*^ Closest placement reference only; ANI reflects phylogenetic distance, not a species-level match.

^*b*^ Genome not assigned to closest species as ANI falls outside the pre-defined species radius.

## Data Availability

The genome assemblies and annotations have been deposited at NCBI GenBank in BioProject PRJNA1333610 and are available through BioSample numbers SAMN51801132 through SAMN51801135 and SAMN56310562 (Table 1). Individual Whole Genome Shotgun project accession numbers are listed in Table 1. The versions described in this paper are the first versions, with the exception of GG3 (JBRIIR000000000.2). Raw sequencing reads have been deposited in the NCBI Sequence Read Archive under accession numbers GG2: SRX31127591, GG3: SRX31127595, and SRX31127594; GG7: SRX31127597 and SRX31127596; GG8: SRX31127599; and GG23: SRX32479453 and SRX32479452 within BioProject PRJNA1333610.
